# Parallel gain modulation mechanisms set the resolution of color selectivity in human visual cortex

**DOI:** 10.1126/sciadv.adm7385

**Published:** 2024-09-11

**Authors:** Marie-Christin Schulz, Mandy V. Bartsch, Christian Merkel, Hendrik Strumpf, Mircea A. Schoenfeld, Jens-Max Hopf

**Affiliations:** ^1^ Otto-von-Guericke University, Magdeburg 39120, Germany.; ^2^ Donders Institute for Brain, Cognition and Behaviour, Radboud University, Nijmegen 6500HB, Netherlands.; ^3^ Kliniken Schmieder, Heidelberg 69117, Germany.; ^4^ Center for Behavioral and Brain Sciences, Magdeburg 39120, Germany.; ^5^ Leibniz-Institute for Neurobiology, Magdeburg 39118, Germany.

## Abstract

Color discrimination is fundamental to human behavior. We find bananas by coarsely searching for yellow but then differentiate nuances of yellow to pick the best exemplars. How does the brain adjust the resolution of color selectivity to our changing needs? Here, we analyze the brain magnetic response in the human visual cortex to show that color selectivity is adaptively set by coarse- and fine-resolving processes running in parallel at different hierarchical levels. Those include a gain enhancement in the higher-lever cortex of color units tuned away from the target to resolve very similar colors and a coarsely resolving gain enhancement in the mid-level cortex of units tuned to the target. Our findings suggest that attention operates on a form of multiresolution representation of color at different levels in the visual hierarchy, which keeps selectivity adaptive to a changing resolution context.

## Teaser

Human color selectivity is adaptively set by coarse- and fine-tuning processes operating in parallel in the visual cortex.

## INTRODUCTION

Visual attention increases the resolution of spatial selection, thereby facilitating or impeding discrimination performance (*1*–*5*). Attention also increases the resolution of feature discrimination by modulating feature-selective cortical units to bias the target representation. Several mechanisms have been reported to be involved. Seminal work in the monkey shows that feature attention increases the gain of feature-selective units coding the target to the extent that they are tuned to the attended feature (*6*–*8*). At the neural population level, such gain enhancement plays out as an increase in feature selectivity, by enhancing the cortical response tuned to the attended feature value and attenuating the response to feature values opposite to the attended one (*9*). Work in humans suggests that feature attention modulates neural gain in a nonlinear way, with target enhancement being combined with stronger attenuation in the immediate surround of the attended feature value (*10*–*12*). Magnetoencephalographic (MEG) research showed that processes of gain enhancement are followed by surround attenuation (SA) to first bias the representation of the attended feature value and then demarcate it from surrounding nontarget values (*13*–*15*).

**Fig. 1.**
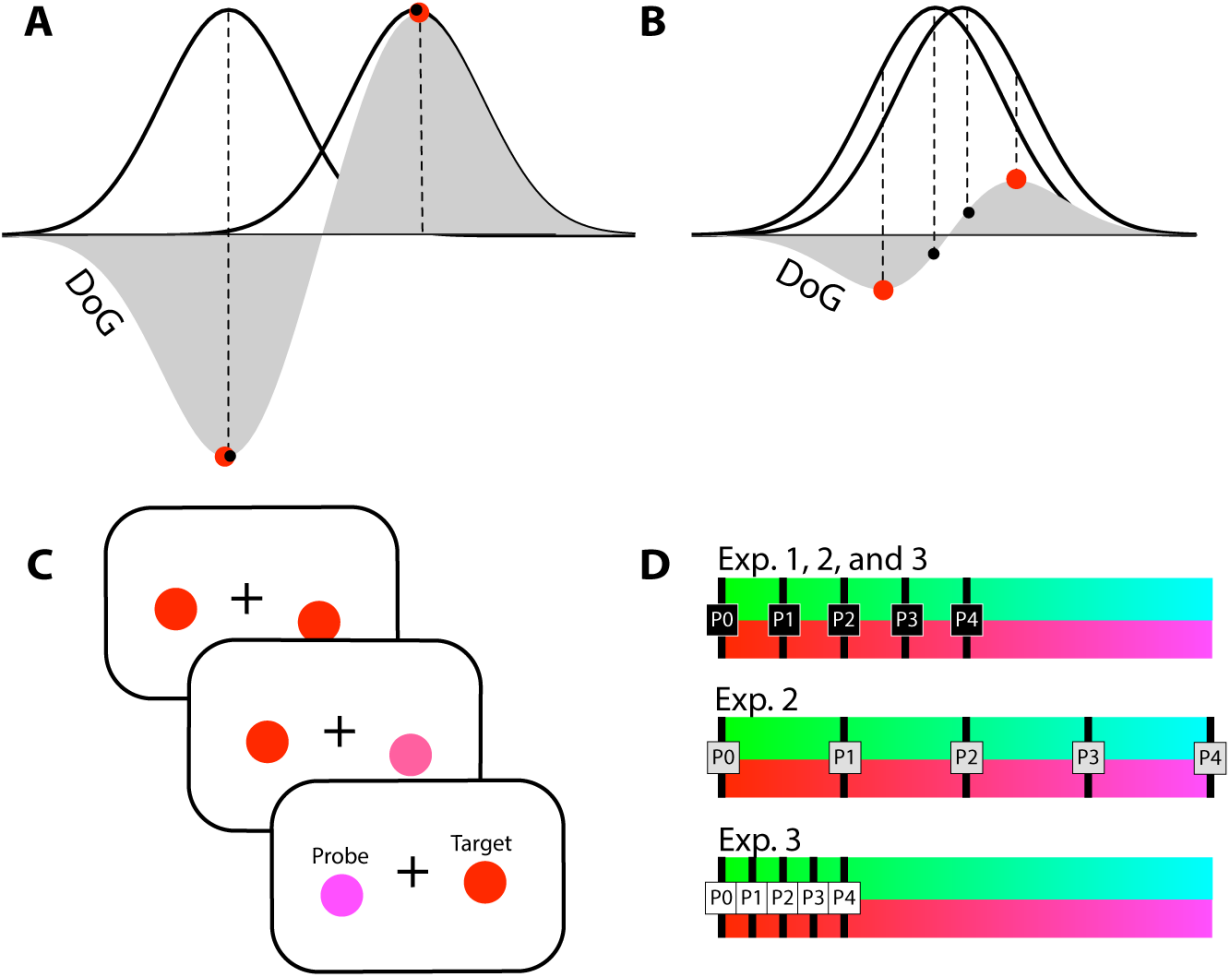
Experimental predictions and stimulus description. (**A**) A situation where tuning functions of units selective for two colors (black solid Gaussians) show minimal overlap, such that the maxima (red circles) of their difference (difference of Gaussians (DoG), gray areas) roughly appear at their tuning maximum (black circles). Attentional gain is best applied to one of those maxima for discriminating those colors. (**B**) A situation where the tuning functions of the to-be-discriminated colors show strong overlap. Here, the DoG maxima are shifted to color values away from their tuning maxima. As units tuned to these color values away show the largest amplitude variation between the colors, attentional gain is best applied here. (**C**) Examples of trials testing the red-purple range. (**D**) Sketch of the relative range of target-to-probe color distances (probe distances) in experiments (Exp.) 1 to 3. The probe distances (P0 to P4) were constructed such that the distance between neighboring colors of the intermediate condition was doubled for the coarse condition (Exp. 2) or halved for the fine condition (Exp. 3).

However, the initial step of increasing the gain of the attended feature value becomes ineffective in situations, where a selection must be made among very similar feature values. For example, selecting a banana among cucumbers requires a coarse differentiation between yellow and green. To judge the ripeness among bananas, however, one needs to discriminate among various greenish yellows. In the latter situation, enhancing the gain of units coding yellow becomes ineffective because this would not differentiate between units with very similar tuning. Figure 1 (A and B) provides an illustration. When tuning functions of units selective to different colors (black solid Gaussians) show minimal overlap as in (Fig. 1A), a gain enhancement of one or the other color would provide the best signal-to-noise ratio (SNR) for identifying a difference. This is because the maxima (red circles) of the response difference (difference of Gaussians, DoG, gray areas) approximately coincide with their tuning maxima (black circles). When the to-be-discriminated colors are very similar as in (Fig. 1B), the tuning functions show considerable overlap. Here, their DoG maxima (the most informative response difference) do not coincide with units most selective for the colors, and attentional gain would be best applied to units showing selectivity at those DoG maxima. Several studies have shown that such a shift of selection bias underlies the fine discrimination of feature values (*16*–*23*).

In daily life, however, the demands of resolving featural differences change constantly. For example, when shopping for bananas, you may want to first find them among all the green vegetables, but then you may want to select the yellower pieces among them—selection steps that require you to switch from a coarse resolution of selection in color space to a much finer one. How the brain accomplishes such changes to a given resolution context is an open question. In the present work, we investigate the cortical processes underlying the adjustment of color selectivity under changing resolution conditions using high spatiotemporal resolution MEG brain recordings. We record the MEG while participants judge whether a color patch that changes from trial to trial matches the color of a reference patch drawn in a pure color (red or green; Fig. 1C). Participants are instructed to compare the changing color to the reference color and tell whether they are the same or different. We refer to the reference color as the (nominal) target and the changing color as the probe color, even though that both colors are attended and targeted on a given trial. The color of the probe varies in five equidistant steps in color space relative to pure red or green toward purple or turquoise, respectively (Fig. 1D). In different experiments and trial blocks, we set the average required resolution of color selectivity to a coarse, medium, or fine level by presenting target-probe pairs that differ in large, medium, or small step sizes from each other, respectively. Brain activity underlying color discrimination is assessed by analyzing the location and time course of cortical current sources, estimated from event-related magnetic fields (ERMF), as a function of the probe-target distance.

## RESULTS

On each trial of all experiments, participants were to report whether the color of circles, one in the left and one in the right visual field (VF), matched or not (Fig. 1C). One circle, randomly appearing in the left or right VF, was always drawn in a pure red or green color (target). The other circle (probe) was randomly drawn from one of five colors equidistantly separating (from zero to four steps away) a section of the color space progressing from red either toward purple or from green toward turquoise, as illustrated in Fig. 1D (cf. fig. S1 for the exact definition of color distances in CIELUV space). Experiment 1 used an intermediate step size in color space. Half of the trial blocks were run as a control condition, where participants were to ignore color and report whether the left or right circle was elevated relative to the other side. Experiments 2 and 3 tested large and small step sizes in color space, with the intermediate step size used in experiment 1 always serving as a within-experiment reference condition.

The general experimental logic is as follows. When performing the control condition where participants are to discriminate the relative elevation of circles, the different target-probe combinations would elicit a similar response, as color is irrelevant and each probe color appears equally frequent. That is, the amplitude profile of the probe’s response as a function of distance to the target would be flat. If, during the color discrimination task, attentional gain is systematically applied to certain points in color space (to facilitate the separation of the target and the probe as illustrated in Fig. 1, A and B), an overall gain bias would build up at those points across an entire experimental block. As the probe colors sample the color range equidistantly in each experimental condition, they will reveal amplitude changes as a function of color distance to the target (the target color is present in each trial). Together, this will finally allow us to assess the particular involvement of gain modulation processes as a function of different color resolution settings.

### Experiment 1 (intermediate resolution)

**Fig. 2.**
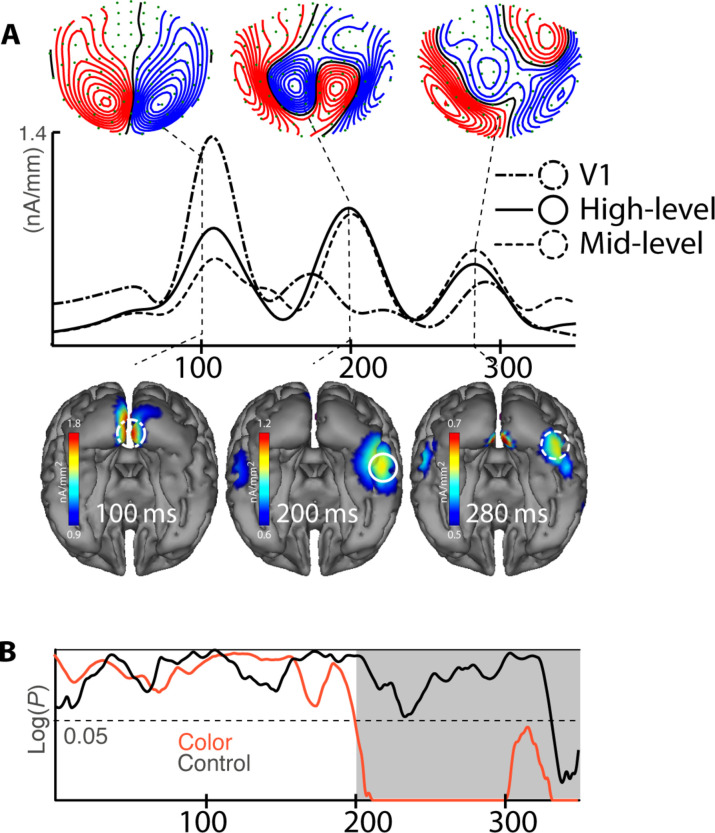
Overall ERMF response (Exp. 1). (**A**) ERMF distribution and current source density (CSD) maps at time points after stimulus onset showing maxima of the overall magnetic response (average over all probe distances of the color condition). The source waves in the middle show the time course of source activity averaged over current dipoles inside the regions of interest (ROIs) centered at the CSD maxima in V1 (dash-dotted line), mid-level (dashed line), and high-level visual cortex (solid line). (**B**) Results of tANOVAs [time course of *P* values, plotted as log(*P*)] testing the effect of probe distance variation on the ERMF response in the color condition (red) and the control condition (black).

Behavioral performance data of all experiments are reported in fig. S2. To first define the overall response to the color stimuli, we analyzed the average ERMF response collapsed over all probe distances of the color condition. ERMF distributions and corresponding current source density (CSD) estimates are displayed in Fig. 2A. The overall color response shows a sequence of three temporal CSD maxima, with the first around 90 to 110 ms located to the primary visual cortex (V1; dash-dotted circle and source wave). The second maximum around 200 ms arises in high-level visual areas (solid circle and source wave). A third maximum appears between 250 and 280 ms in the mid-level visual cortex (dashed circle and source wave). The source waves (time course of CSD estimates) are taken from regions of interest (ROIs; circles) at the CSD maxima. The sequence of source maxima displays an initial forward propagation of activity from the primary to higher-level visual cortex, followed by a small delay in mid-level areas. To identify cortical activity variation due to differences in probe distance, we performed a time-sliding topographical analysis of variance (tANOVA; cf. Materials and Methods for details) in a time range of 0 to 350 ms after stimulus onset. Figure 2B shows the results for the color (red trace) and the control condition (black trace). As visible, the magnetic field response of the color condition is significantly (*P *< 0.05) modulated by probe distance starting around 200 ms after stimulus onset. The initial response around 100 ms in V1, however, does not vary with probe distance. In the control condition, probe distance does not modulate activity in the visual cortex at all during the first 300 ms, which is consistent with color differences being irrelevant in this condition. A CSD analysis of the control condition is provided in fig. S3.

**Fig. 3.**
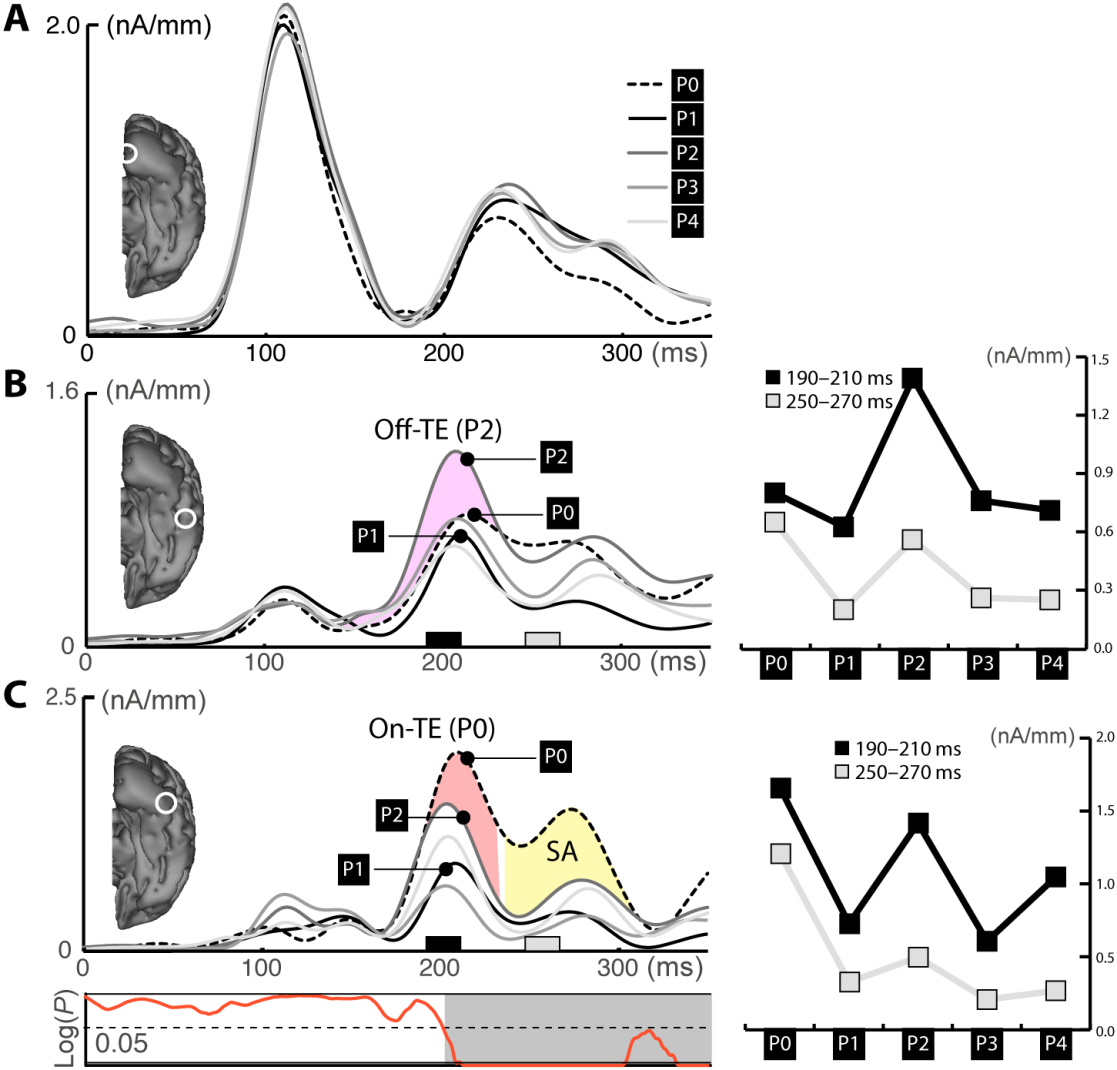
Intermediate resolution (Exp. 1). (**A**) Source waves of the five probe distance conditions from the V1-ROI defined in the overall analysis. (**B**) Source waves of the ROI in high-level visual cortex. The Off-TE effect is visible as an enhanced response of P2 and is highlighted by the purple area between P2 and P0. The diagram on the right plots the mean amplitude of source activity in an early (black) and later time window (gray) for each probe distance. (**C**) Source waves of the mid-level ROI. The On-TE effect is visible as an enhanced response of P0 and is highlighted by the red area between P2 and P0. The yellow area highlights the SA effect. The diagram below the source waves replots the tANOVA results of the color condition shown in Fig. 2.

To assess the cortical activity as a function of probe distance, CSD estimates were computed separately for each of the probe distance conditions. Figure 3 displays the source waves for distances P0 to P4 in ROIs placed at the temporal maxima of the overall response (Fig. 2A). The diagrams on the right summarize amplitude measures in an early (190 to 210 ms, black) and late (250 to 270 ms, gray) time range in those ROIs. In the primary visual cortex (Fig. 3A), we see a strong initial response to the color stimuli, but source activity does not vary as a function of probe distance. In the higher-level visual cortex (Fig. 3B), we see strong source activity for P2 between around 200 ms as compared to all other conditions including the target color, as highlighted by the purple area between source waves of P0 and P2. This indicates that color selection at this cortical level is initially driven by a gain enhancement not coinciding with the target. In the mid-level ROI (Fig. 3C), instead, the source activity is largest for the target color (P0) and smaller for P2 (highlighted by the red area). All other probe distances show even smaller responses, indicating that on-target enhancement (On-TE) dominates the response at this level, with Off-TE appearing to a lesser degree. Hence, color selection starts with two different gain modulation processes running in parallel in higher-level (Off-TE) and mid-level cortex (On-TE). In the late time window (250 to 270 ms), the source activity of all probe conditions, including P2, is attenuated, leaving only P0 with a strong response (yellow area). This delayed amplitude reduction of all colors except for the target reflects SA in color space as documented previously (*14*).

### Experiment 2 (coarse resolution)

Under the intermediate resolution settings of experiment 1, source activity in the visual cortex shows a sequence of modulation patterns, with parallel Off-TE in the higher-level cortex and On-TE in the mid-level visual cortex, followed by SA in the mid-level cortex. Under coarser resolution conditions, however, where tuning functions show much less overlap (Fig. 1A), Off-TE would not be beneficial. Instead, a gain amplification of the target color alone would increase SNR sufficiently. Experiment 2 addresses this prediction by doubling the step size in color space relative to experiment 1 (cf. Fig. 1D).

**Fig. 4.**
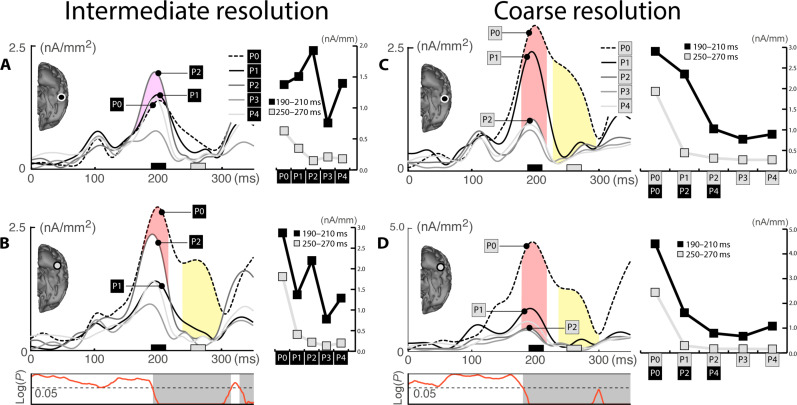
Intermediate and coarse resolution condition (Exp. 2). Source waves of the high-level (**A**) and mid-level ROI (**B**) of the intermediate resolution condition. The Off-TE and On-TE effects are highlighted in purple and red, respectively. The later SA effect is shown in yellow. The diagram at the bottom shows the results of a tANOVA testing the effect of probe distance variation at each subsequent time sample between 0 and 350 ms. (**C** and **D**) Source waves of the high-level (C) and mid-level ROI (D) of the coarse resolution condition.

Source waves for the five probe distance conditions of the intermediate resolution condition are shown in Fig. 4 (A and B), which replicate the observations in experiment 1 almost perfectly. Around 200 ms, we see a prominent source maximum for P2 relative to all other conditions (purple area in Fig. 4A) in the higher-level cortex (Off-TE). In the mid-level cortex, in contrast, P0 shows the largest response (red area in Fig. 4B), indicating that On-TE dominates the response pattern. In a later time range beyond 250 ms, we see SA (yellow area). That is, all probe distances are strongly attenuated except for P0, which remains at a high response amplitude until ~300 ms.

The coarse resolution condition (Fig. 4, C and D) shows a very different pattern. There is no sign of an Off-TE effect in the higher-level visual cortex. Instead, the initial response (around 200 ms) is maximal for P0 followed by P1 (red area), with P2 to P4 showing only minimal activity enhancements (Fig. 4C). In a later time range (250 to 300 ms), the response of P1 is strongly attenuated, leaving only P0 with a substantial response amplitude (yellow area). In the mid-level cortex (Fig. 4D), we see a strong response bias for P0 very early on, with P1 showing little enhancement relative to the remaining probe distances. Again, in the late time range beyond 250 ms, all probe distances, except P0, are strongly attenuated. Hence, the source activity pattern of the coarse resolution condition suggests an initial bias for the target color in both the higher- and mid-level cortex (On-TE), followed by SA demarcating the target color.

### Experiment 3 (fine resolution)

Experiments 1 and 2 revealed that when participants set their expected color resolution to an intermediate level, discrimination starts with Off-TE in the higher-level and On-TE in the mid-level cortex, followed by SA. As expected, Off-TE does not appear under the coarse resolution settings in experiment 2, consistent with the interpretation that Off-TE does not help increase the SNR of discrimination between very different colors (Fig. 1A). If this is the case, then one would predict that when increasing the required color resolution (fine condition) relative to the intermediate conditions of experiments 1 and 2, discrimination would rely on Off-TE even more. This is because the number of trials with a small target-probe distance is larger, and the amplitude of the DoG maximum (as illustrated in Fig. 1B) decreases with increasing overlap of the tuning functions, thereby requiring more attentional gain to amplify the response difference for a sufficient SNR. Experiment 3 investigates these predictions.

**Fig. 5.**
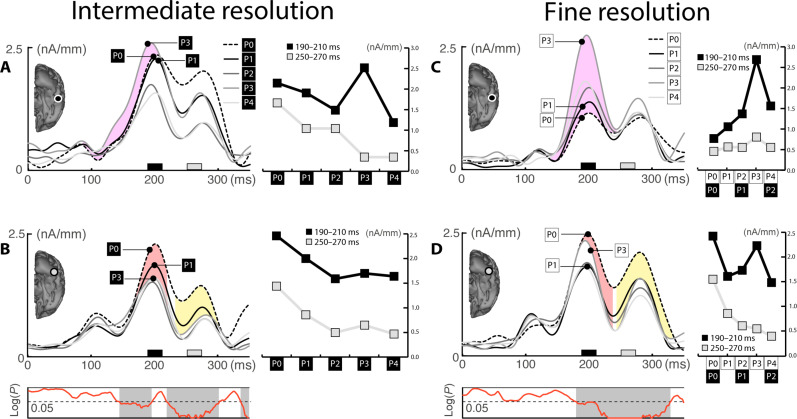
Intermediate and fine resolution condition (Exp. 3). Source waves of the high-level (**A**) and mid-level ROI (**B**) of the intermediate resolution condition. The colored areas highlight the amplitude difference between P3 and P0. The diagram at the bottom shows the results of a tANOVA testing the effect of probe distance variation at each subsequent time sample between 0 and 350 ms. (**C** and **D**) Source waves of the high-level (C) and mid-level ROI (D) of the fine resolution condition.

Figure 5 (A and B) shows source waves for the five probe distance conditions of the intermediate condition. Consistent with experiments 1 and 2, we see a selective Off-TE in the higher-level cortex around 200 ms (Fig. 5A, purple area), albeit for P3 and not P2. In the mid-level cortex (Fig. 5B), instead, the response pattern suggests On-TE (red area), with P0 showing the largest initial response, that gradually falls off for P1 and P2. In a later time window, however, responses of nontarget colors become attenuated relative to P0 (yellow area). Hence, as in experiments 1 and 2, the intermediate resolution condition is initially associated with Off-TE and On-TE at different levels in the cortical hierarchy, followed by SA.

For the fine resolution blocks (Fig. 5, C and D), we find a strong response enhancement for P3 around 200 ms (Off-TE) in higher-level cortex relative to all other probe distances (Fig. 5C), with P0 eliciting the smallest response. The Off-TE effect of the fine condition relative to P0 (P3-to-P0 ratio = 2.4) is substantially larger than the Off-TE effect of intermediate conditions of experiments 1 to 3 [mean P2(3)-to-P0 ratio = 1.41]. In the mid-level cortex, we see an initial increase for P0 and a slightly smaller enhancement for P3 around 200 ms (On-TE), followed by SA of all probe distances relative to P0. The strong initial Off-TE for P3 in the higher-level cortex confirms that under fine resolution conditions, a gain shifted away from the target color is emphasized. Notably, P3 of the fine resolution blocks corresponds with a probe distance in-between P1 and P2 of the intermediate blocks (cf. Fig. 1D), suggesting a smaller absolute shift of gain away from the target. This is consistent with the DOG maximum of tighter overlapping tuning functions getting closer to the target-probe range.

Experiment 3 replicates the presence of an Off-TE maximum for the intermediate condition seen in experiments 1 and 2. This maximum, however, appears at P3 and not P2—a shift that implies that participants set their resolution depending on the overall context in a given experiment. (A further illustration of the influence of the overall experimental context is provided in fig. S4, where we compare the response to identical target-probe pairs across all experiments.) But why did the Off-TE maximum shift to P3 in experiment 3? The smallest distance is likely critical for setting the resolution bias in an experiment. In experiments 1 and 2, P1 of the intermediate resolution is the smallest distance overall. In experiment 3, however, the fine condition defines the smallest distance. It has been shown that color attention to small color distances warps the perception of color space such that smaller distances are experienced to be larger (*22*), which may shift the Off-TE maximum. Of course, this tentative possibility requires further experiments for clarification.

## DISCUSSION

The results of the three experiments show that participants solicit different cortical modulations in a flexible way to adjust the expected resolution of color discrimination. The adjustment involves a sequence of cortical operations unfolding in different combinations between ~200 and 300 ms in higher-level and mid-level extrastriate, but not in the early, visual cortex (V1). In a coarse resolution context (experiment 2), the sequence starts with a gain enhancement for the target in the higher-level cortex, followed by a sharpening of selectivity via SA. This sequence of modulations reconciles with previous work on color-based attention where participants were to discriminate among cardinal colors requiring a coarse resolution (*14*). When the discrimination is set to intermediate or high resolution in color space (experiments 1 to 3), the sequence starts with parallel Off-TE and On-TE in the higher-level and mid-level cortices, respectively. Independent of the resolution context, these early processes are followed around 250 ms by a gain attenuation of color values surrounding the target consistent with the ST account (*12*, *13*). When the required resolution of selectivity increases, the Off-TE modulation becomes stronger and shifts to a color value closer to the target that better resolves the color difference. Last, a comparison across experiments reveals that the Off-TE bias changes depending on the overall resolution settings of an experiment. This suggests that the cortex adjusts color selection processes adaptively in relation to a general context to facilitate selectivity.

### Multiple modulation processes combine to set the resolution of color discrimination

Overall, the present results add to previous work showing that, at the neural population level, feature attention can operate via several mechanisms, including a bias of the target as proposed by the feature similarity gain model (*9*, *24*, *25*), a bias away from the target as in accounts referred to as optimal gain or tuning (*16*–*23*), and via selective tuning (*10*–*12*, *14*). Furthermore, they dovetail with behavioral observations indicating that feature attention operates by combining gain enhancements and the sharpening of tuning of the population’s response (*26*). We show how these cortical mechanisms combine to adapt discrimination performance to a particular resolution in a flexible manner. The most notable finding is that for setting a higher resolution of discrimination in color space, the visual cortex resorts to a combination of two attentional processes (Off-TE and On-TE) running in parallel at different hierarchical levels in the ventral extrastriate cortex. Those precede processes of selective tuning, ultimately demarcating the target via SA, by roughly 50 ms. Under the highest resolution conditions, Off-TE is emphasized as a selection process, whereas the lowest resolution condition involves target gain only (On-TE). This indicates that participants adjust their color selectivity to the expected resolution and that this is done by adaptively weighting involved modulation processes to attain optimal SNR for target selection.

### Color attention operates on different resolution scales

**Fig. 6.**
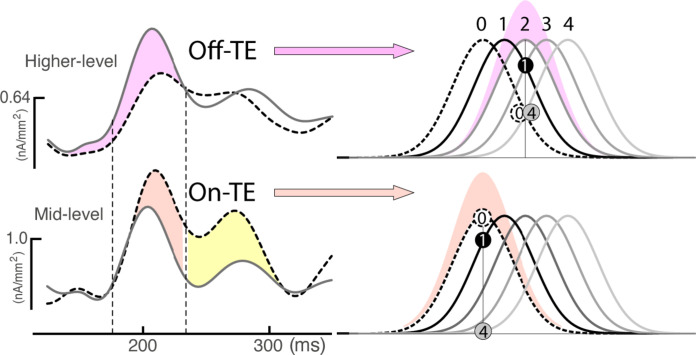
Parallel tuning mechanisms. The waveforms on the left replot the source waves elicited by P0 and P2 in Fig. 3 (A and B). Feature attention operating via parallel Off-TE and On-TE in the visual cortex allows to accommodate fine and coarse discrimination processes in a flexible way. An attentional process just operating via Off-TE at color 2 (purple Gaussian) would facilitate the discrimination of very similar colors like 0 and 1 but would fail on very different colors like 0 and 4, to which units tuned to 2 would respond with the same amplitude. In addition, a parallel-running On-TE process at 0 (red Gaussian) would simultaneously gauge the difference between 0 and 4, such that overall discrimination remains effective and at an optimal SNR, no matter whether a large or small color difference is encountered on a given trial.

As On-TE cannot resolve small distances in color space, why does it appear under intermediate and high-resolution conditions? We propose that the parallel operation of Off-TE and On-TE in the visual cortex is analogous to attention modulating a multiresolution representation (*27*, *28*) in feature space. This is beneficial in the present experimental context as it enables to resolution of small distances in color space while simultaneously retaining the selectivity for larger distances. As illustrated in Fig. 6, the Off-TE process alone would facilitate the resolution required on trials with small color distances but would fail on trials with larger distances. Say, on trials displaying colors 0 and 1, a gain increase of units tuned to a color two steps away (color 2, purple area) from the target (color 0) would be optimal. This separates colors 0 and 1 better than the gain applied to color 0 (red area). However, on trials presenting colors 0 and 4, color 2 units would respond with the same amplitude. A gain enhancement here would not help to discriminate against them. Instead, a gain enhancement of color 0 would facilitate the discrimination. Hence, the combination of Off-TE and On-TE allows us to flexibly gauge smaller and larger color separations on trials within a given resolution context. Consistently, with increasing resolution, more emphasis is given to the Off-TE process to accommodate smaller distances. The two processes operate like quasi-parallel attentional filters that are given variable emphasis to set the optimal resolution of selectivity. Note that the mechanism is conceptually analogous to what has been proposed to underly the increase of attentional resolution in spatial tasks (*1*, *2*, *29*, *30*).

### Alternative accounts of the modulation maximum away from the target

Here, we propose that Off-TE reflects an overall shift of selectivity away from the target toward a range maximizing the SNR of discrimination of small color differences (Fig. 1B). This explanation has conceptual overlap with a mechanism referred to as optimal gain or tuning, which holds that the representation of a target-defining feature in visual search shifts in a direction that enhances target-to-distractor disparity (*17*, *21*, *23*). A shift of selectivity is also predicted by an account referred to as relational tuning (*31*–*33*), according to which selectivity is biased toward relative properties of a search target that best demarcates it from distractors. For example, for selecting a reddish purple among red items, attention would bias any bluer item in the red-blue range, thereby shifting selectivity toward the blue end. Such relational bias is sensible when a target must be selected against irrelevant distractors, as in a visual search. In the present experiments, both the target and the probe are effectively targets. For relational tuning to account for the parallel On-TE and Off-TE effects, the mechanism would have to be flexible such that it emphasizes the target end of the color range on large but the probe end on small color distances.

Last, the Off-TE effect could be caused by a selection process unrelated to a shift in selectivity, as discussed above. For example, it is conceivable that it reflects the color distance where observers become confident in discriminating the probe from the target. The behavioral performance data of experiment 1 (fig. S2) seem compatible with such a possibility, as we see a sharp increment in discrimination accuracy between P1 and P2. However, in experiments 2 and 3, this performance increment appears already between P0 and P1, and in experiment 3 (intermediate condition), accuracy is very high at P1 while Off-TE appears at P3, rendering an explanation in terms of discrimination success unlikely.

A related possibility worth considering is that P2 of the intermediate resolution condition may be at or close to a point in color space where participants would experience a change of the categorical representation (e.g., red-to-purple border). The Off-TE could then simply reflect a stronger cortical response close to the categorical border. A short color categorization task that was run after experiment 1 revealed that the red-purple and green-turquoise borders lie between P1 and P2 (cf. fig. S1). If Off-TE reflects proximity to those borders, then an amplitude enhancement would be expected to appear for both P1 and P2. Furthermore, in experiment 2, P1 of the coarse condition is closest to this border but does not show the response maximum. Last, in experiment 3 (intermediate resolution condition), the Off-TE maximum appears significantly away from this border at P3. Hence, the Off-TE is unlikely to be caused by proximity to the experienced red-purple and green-turquoise borders. Nonetheless, given that attention can change the way we perceive color (*22*), we cannot rule out that attention-driven adjustments of the resolution of color selectivity also change categorical borders.

## MATERIALS AND METHODS

### Participants

Thirty-six participants took part in experiment 1. Thirty-two participants each took part in experiments 2 and 3. After artifact rejection (see the “Primary MEG data analysis” section), 28 participants (14 female, mean age 28, 1 left-handed) remained for the first experiment and 26 for experiment 2 (18 female, mean age 27) and experiment 3 (15 female, mean age 27, 2 left-handed). All participants gave written informed agreement and were paid for participation (€8/hour). All participants reported having normal or corrected-to-normal vision and neurologically normal status. The experimental procedures were approved by the ethics board (study approval number: 141/20) of the Otto-von-Guericke University, Magdeburg, Germany.

### Stimulus presentation

The stimuli were presented via back-projection onto a semitransparent screen using a DLP-LED-projector (ProPixx, VPixx Technologies Inc., Saint-Bruno, QC, Canada; resolution: 1920 × 1080; refresh rate: 120 Hz). The back-projection screen was placed inside the electromagnetically shielded recording chamber (μ-metal; Vacuumschmelze). The viewing distance to the participant’s eyes was 1.0 m. The stimulus presentation was coordinated by Presentation software (Neurobehavioral Systems Inc., Berkeley, CA, Version 18.3). Responses were given via button press of the right index and middle finger with a LUMItouch response system (Photon Control Inc., Burnaby, DC, Canada).

### Stimuli

Each trial presented a circular color target in one VF and a circular color probe in the opposite VF (each with a diameter of 3.1° of vision, the center placed 4.9° to the left and right, respectively, and 3.1° below fixation in the lower VF; cf. Fig. 1C). The color target randomly appeared in the left and right VF and was always drawn in a pure red or green. The probe color randomly varied from trial to trial among five equidistant steps in color space (see the “Definition of colors” section), either ranging from red to purple or from green to turquoise (zero to four steps away from the target). Both the target and probe colors are task-relevant and attended, such that they can be considered targets. Nonetheless, we refer to the pure color patch as the nominal target because it appears in every trial and participants are instructed to compare the changing color to this patch. Three different resolution contexts were defined: intermediate (in all experiments), coarse (in experiment 2), and fine (in experiment 3) by using target-probe pairs that differ in intermediate, large, or small step sizes from each other (see Fig. 1D for a schematic depiction). In addition, the patches were vertically offset relative to each other by 0.86°, with either the patch in the LVF or the RVF being elevated on a given trial, which served to construct a control condition in experiment 1. The offset was kept constant across all experiments and conditions. Stimulus frames appeared trial by trial for 295 ms with a randomly varying interstimulus interval of 1000 to 1200 ms, where only the fixation cross was presented (rectangular distribution).

### Procedure

The participants fixated a permanently visible gray cross in the center of the screen (RGB value: 150, 150, 150) on a dark gray background (RGB value: 40, 40, 40). They were instructed to attend both color patches and to report whether the colors matched or not with a two-alternative button press (index or middle finger) of the right hand. Specifically, participants were told that a pure color (red or green), referred to as the target, would always appear on a given trial and that the performance strategy would be to compare the other (changing) color to the target. The mapping of the index and middle finger onto the match and non-match response was counterbalanced across participants. Experiment 1 used a control task, in which the participants were to ignore the colors and report with a two-alternative button press of the right hand (index or middle finger) which the color patch was elevated relative to the other side. During the experiment, no performance feedback was given. In each experiment, participants performed a total of 12 trial blocks resulting in a total of 2160 trials. Each block lasted ~5 min and contained 180 trials [every 30 trials, there was a short pause, in which participants were encouraged to blink, as well as reminded of the current resolution context (experiments 2 and 3)]. The blocks started with a short instruction about finger mapping, the current task, and the current resolution context (experiments 2 and 3). The color ranges (red-purple and green-turquoise) changed from block to block, with the task (experiment 1) or the color resolution context (experiments 2 and 3) changing every second block. Participants performed six blocks for each color range and experimental condition. This yielded a total of 108 trials for each target-probe color combination of a given experimental condition. Each block contained 36 matches and 144 non-matches. The block order of color ranges (red-purple and green-turquoise) and resolution settings (fine, intermediate, and coarse) was counterbalanced between participants.

### Definition of colors

The color gamut possible to be projected with the ProPixx system was first defined in HSV. Then, the two color ranges for testing (red-purple and green-turquoise) were defined in HSV by starting at the pure target colors red (360°) and green (120) and ending 60° apart at a purple (300°) and a turquoise (180°) color. These ranges were then divided into 2° color steps, resulting in 31 colors, which were then set to equal luminance (in ProPixx) at approximately 30 cd/m^2^. Equal luminance was afterward confirmed by spectrometer measurements (CRS SpectroCal spectroradiometer, Cambridge Research Systems).

P0 to P4 of the intermediate resolution condition (experiments 1 to 3) were defined in the following way. After defining the set of 31 colors in HSV and determining their coordinates in CIELUV space, four roughly equidistant values away from pure red or green in uv space were chosen to define the five target-probe color combinations of the intermediate resolution condition (cf. fig. S1). The target-probe combination with the same pure color was named P0. The target-probe combination with the pure target color and the most distant probe color was referred to as P4. Then, P2 was chosen by halving the range between P0 and P4. Afterward, P1 and P3 were chosen by halving the range between P0 and P2 as well as between P2 and P4, respectively.

For the fine resolution condition (experiment 3), the step size was halved relative to the intermediate condition by dividing the P0-to-P2 range into four steps, such that P2 of the intermediate condition became P4 of the fine condition and P1 of the intermediate condition became P2 of the fine condition. The probe colors of P1 and P3 were finally defined to lie halfway between their neighboring colors.

For the coarse resolution condition (experiment 2), the colors were defined as follows: The first two step sizes were increased by skipping every second probe color of the intermediate resolution condition, such that P2 and P4 of the intermediate condition became P1 and P2 of the coarse resolution condition, respectively. P3 and P4 were then defined by further continuing toward purple or turquoise in roughly equal step sizes. Note that in both color ranges, the steps defining P3 and P4 were set to a somewhat smaller distance than those defining P1 and P2. This was done to keep stimulus luminance at ~30 cd/m^2^. Because of technical reasons, the possible luminance drops substantially below this value for colors closer to blue.

### Definition of categorical borders (experiment 1)

After finishing the MEG recording session in experiment 1, every participant was asked to categorize the colors used in the MEG experiment in a short behavioral experiment (13.5 min). The latter served to determine where participants would place the categorical border within the red-purple and green-turquoise ranges. On each trial, two color patches drawn in target-red and the most distant purple (target-green and the most distant turquoise) were shown as a reference for 650 ms. Thereafter [interstimulus interval (ISI) of 400 ms], a single color patch appeared for 300 ms and participants were asked to classify it as red or purple (green or turquoise) with a two-alternative button press of the right index or middle finger. Participants performed two trial blocks per color range, each containing 50 trials, resulting in a total of 20 presentations of each color. The categorical border was defined to lie between neighboring colors being more frequently reported as red versus being more frequently reported as purple (green versus turquoise). The experiment revealed that in both the red-purple and the green-turquoise ranges, participants placed the categorical border between P1 and P2 (cf. inset in fig. S1).

### MEG recordings

The MEG data were recorded continuously using an Elekta Neuromag TRIUX triple sensor system with 102 magnetometers (Elekta Neuromag, Oy, Helsinki) inside a μ-metal–shielded recording room (Vacuumschmelze, Hanau, Germany). The head position of the participants inside the dewar was ensured via small foam cushions. During the measurement, contact with the participants was ensured by a communication system (camera, microphone, and speaker). Vertical and horizontal eye movements were registered simultaneously by recording the electrooculogram (EOG) using a unipolar electrode placement beneath the right eye (vertical EOG) and bipolar electrode placements at both eyes’ outer canthi (horizontal EOG). The MEG data were digitized with a sampling frequency of 1000 Hz. MEG and EOG data were bandpass-filtered online from 0.03 to 330 Hz. A photodiode fed into an analog channel of the MEG system was used to check the timing of the stimuli. Response accuracy, response time, and stimulus event codes were recorded and stored alongside the MEG data by Presentation software (Neurobehavioral Systems Inc., Berkely, CA, Version 18.3). In addition to MEG, the electroencephalogram (EEG) was recorded from the scalp with 32 electrodes. Those data are not reported here.

Individual landmarks (inion, nasion, vertex, and left and right preauricular point) as well as an individual three-dimensional model of the skull were digitized (the Polhemus 3SPACE FASTRAK system; Polhemus, Colchester, VT, USA) together with five localizer coils placed at standardized positions (inion, nasion, left and right preauricular point, and vertex) of an EEG cap (Easycap, Herrsching, Germany). The position of the localizer coils was measured in between trial blocks to correct for head movements by spatially realigning the data using MaxFilter.

### Primary MEG data analysis

First, the continuously recorded data were subjected to Maxwell filtering (MaxFilter Software, Elekta Instrument Ab Stockholm, Sweden), for down-sampling the data from 1000 to 500 Hz, to eliminate spatial and spatiotemporal interferences via spatiotemporal signal space separation and to correct for head movements during the measurement. The latter was based on the signal of localizer coils which was recorded intermittently between the trial blocks. Participants with head movements larger than 10-mm vertical translation and horizontal rotation larger than 15° were excluded from further data analysis. Following MaxFilter, the data were visually inspected using FieldTrip (Version 20160419, Radboud University, Nijmegen, Netherlands) under MATLAB (Version 9.7, MathWorks Inc., Natick, MA, USA).

The continuous MEG and EOG data were cut into epochs offline spanning from 0.5 s before until 1 s after stimulus onset. Trials with double responses and responses outside a time window of 200 to 1300 ms after stimulus onset were excluded from further analysis. For artifact rejection, maximum amplitude responses in each epoch and sensor were determined and *z*-transformed. Trials with artifacts were rejected according to individually defined thresholds (cutoff *z* values) for each participant. Thresholds were iteratively adjusted until all major artifacts (like muscle, blinks, and channel jumps) were eliminated from the data. Participants with less than 70% of the trials remaining after artifact removal were excluded from further analysis. For the remaining participants, an average of 15% of trials were rejected. Trials were then averaged selectively for each probe distance, color resolution condition, and participant (relative to a 200-ms baseline before stimulus onset). Data were finally averaged over participants to derive grand average responses.

### Statistical data validation

#### ERMF data

ERMF changes reflecting differences in probe distance were statistically validated using one-way (five-level) temporal analyses of variance (implemented in Curry 8.3) (*34*) based on all sensors (102 magnetometers). Those were computed in a time range between 0 and 350 ms after stimulus onset, separately for each experimental condition. Before the analyses, the data were low-pass filtered at 200 Hz. To account for the problem of multiple comparisons, we followed the approach of Wagner and colleagues (*35*) to use spectral parameters of the data (sampling frequency, 500 Hz; filter frequency, 200 Hz) which yielded a corrected alpha-level of 0 = 0.042 (uncorrected 0.05-level, dashed horizontal lines in Figs. 2 to 5).

#### Behavioral responses

Response accuracy (% correct responses) was statistically evaluated using MATLAB (MathWorks Inc., Natick, MA, USA). Only answers within 200 to 1300 ms after stimulus onset were used to exclude anticipatory and delayed answers. Statistical validation was done using one-way (five-level) repeated-measures ANOVAs. Violations of sphericity were adjusted for on the basis of the Greenhouse-Geisser epsilon. Corrected *P* values are reported.

### Current source analysis

Current sources were estimated using a distributed source model [minimum norm least squares (MNLS) method as implemented in Curry 8.3, Compumedics Neuroscience] with a fixed (surface-normal) dipole orientation. Estimates were computed for each subsequent time sample between 0 and 350 ms after stimulus onset, with noise estimates taken from the baseline period (−200 to 0 ms). Regularization used the χ2 criterion, with the assumption that Δ2 is in the range of data noise. Estimates were computed for the grand average data across participants, using a gray matter segmentation of the MNI brain as source compartment (provided as a standard brain in Curry 8.3). For visualization purposes, the CSD time courses (source waves) were smoothed with a sliding average filter (window width, 20 ms).
