# Supplementary material for: Parallel gain modulation mechanisms set the resolution of color selectivity in human visual cortex
Source: Sci Adv. 2024 Sep 11;10(37):eadm7385. doi: 10.1126/sciadv.adm7385 (PMC11389780; doi:10.1126/sciadv.adm7385)
Supplement: Supplementary file 1 — Figs. S1 to S4 [file sciadv.adm7385_sm.pdf]

Science Advances
AAAS

Supplementary Materials for

Parallel gain modulation mechanisms set the resolution of color selectivity in human visual cortex

Marie-Christin Schulz *et al.*

Corresponding author: Jens-Max Hopf, jens-max.hopf@med.ovgu.de

Sci. Adv. **10**, eadm7385 (2024)
DOI: 10.1126/sciadv.adm7385

This PDF file includes:

Figs. S1 to S4

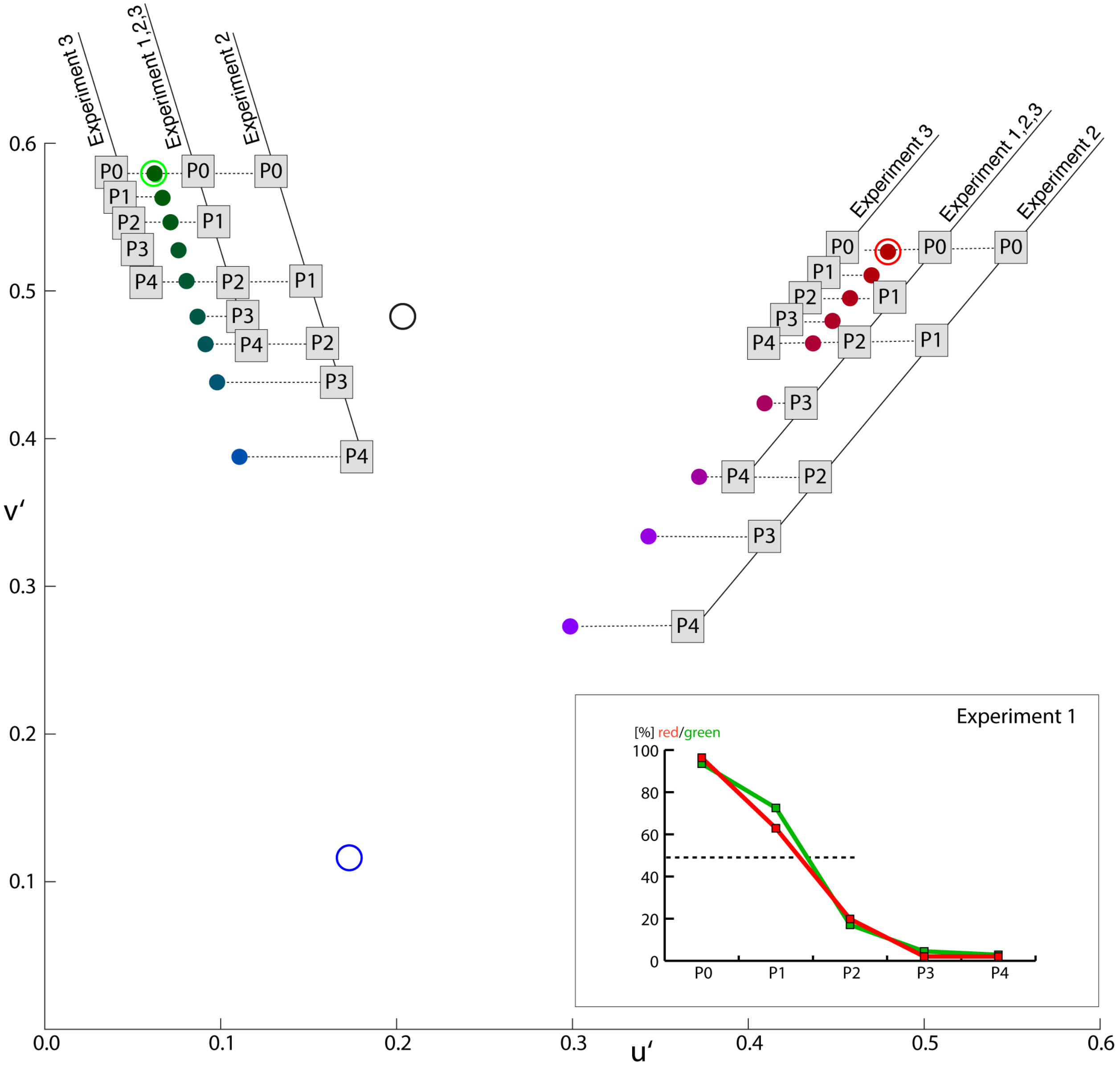

Fig. S1.

Definition of probe-target color distances in CIELUV-space. The filled color circles mark the position of nine colors defining the color-distances in each experiment along the green-to-turquoise and red-to-purple range on a luminance plain of ~30 cd/m^2. The red, blue, and green circles mark the points of pure red, blue, and green in the projector gamut. The black circle highlights the white point. The hue values were placed a the maximum chroma achievable with the projector gamut. Equal luminance was verified with a spectroradiometer (CRS SpectroCal, Cambridge Research Systems). In each color range, color-distances (P0 to P4) were defined relative to pure red or green which served as target colors. **(Inset) Color-categorization task of experiment** 1. Plotted is the proportion (grand-average over subjects) of red (green) answers as a function of the probes' distance to the target-red (target-green). While P1 was more frequently classified as red (green), P2 was more often reported as being a purple (turquoise), indicating that in both color ranges the categorical border is placed between P1 and P2.

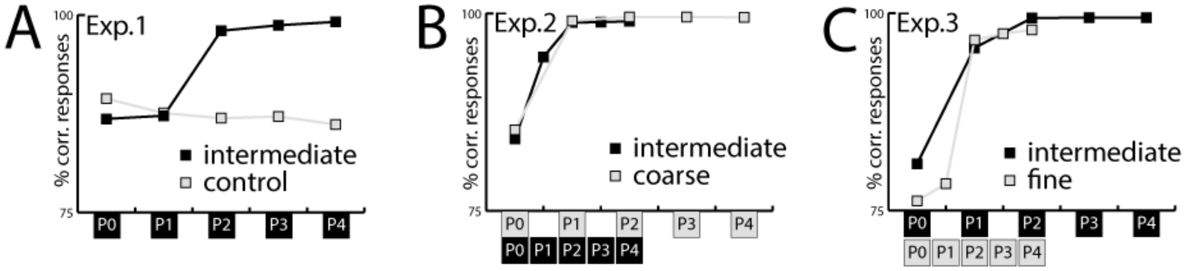

Fig. S2.

<u>Behavioral</u> <u>performance</u> <u>data</u>. Accuracy was above 75% in all experiments, indicating that participants were able to discriminate the color stimuli well under all experimental (color) conditions. **(<u>A</u>)** *<u>Experiment</u> <u>1.</u>* Performance accuracy (% correct responses) on the color condition was above 86% in all probe-distance conditions and increased significantly for larger distances to almost 100% ($F[4,27]=18.64$, $p<0.0001$). For the control condition, the performance pattern is different. Instead of a performance increase with probe-distance, there is a slight, but significant decrease ($F[4,27]=10.66$, $p<0.0001$). **(<u>B</u>)** *Experiment 2*. Accuracy increases as a function of probe-distance in both experimental conditions (coarse: $F[4,25]=58.1$, $p<0.0001$, intermediate: $F[4,25]=47.3$, $p<0.0001$). The coarse condition approaches ~100% at all probe distances except for P0. The intermediate condition shows higher accuracy at P1 relative to P2, and reaches ~100% at P2. **(<u>C</u>)** *Experiment 3*. Accuracy increases with probe-distance in both experimental conditions (fine: $F[4,25]=54.8$, $p<0.0001$, intermediate: $F[4,25]=54.6$, $p=0.0001$). Consistent with P1 showing a very small color difference between the target and probe, accuracy is similar to P0. For P2, however, accuracy approaches 100%. A notable observation in all experiments is that for the probe-target match (P0), performance accuracy is comparably low. This is particularly peculiar in the coarse condition of experiment 2 where the color match should be easily detectable and performance be comparable between P0 and P1. The most likely explanation of this performance drop at P0 is that subjects developed some bias for the non-match response which had to be given on 80% of the trials. On the lesser frequent match-responses this bias had to be broken, which caused the overall response delay.

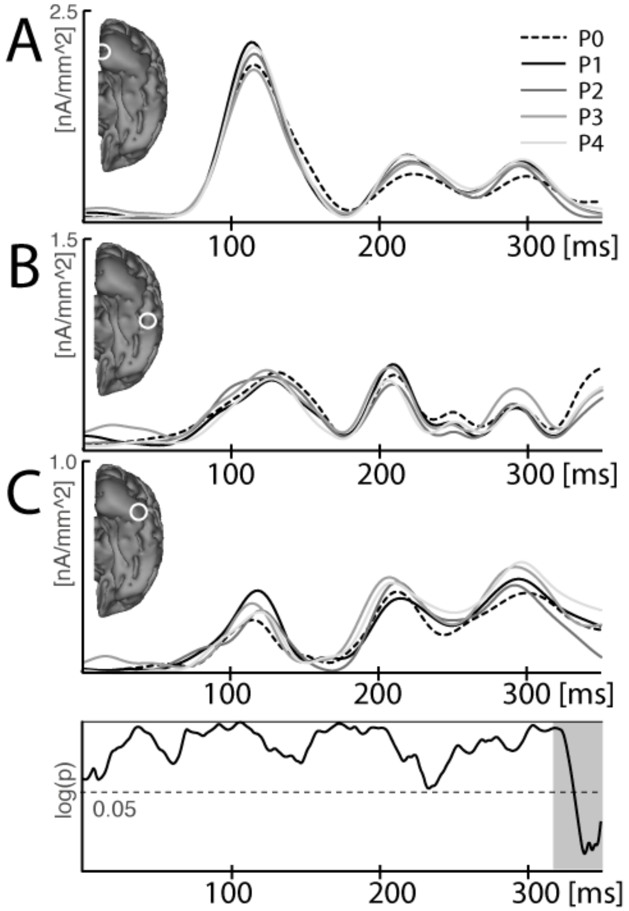

Fig. S3.

Source analysis of control condition (Experiment 1). Source waves taken from ROIs defined by the color condition in V1 (**A**), in high-level cortex (**B**), and mid-level cortex (**C**). As can be seen, there is no significant color variation in the control condition up to 300ms (see results of the tANOVA plotted below (time course of p-values, testing the effect of probe-distance variation on the ERMF response).

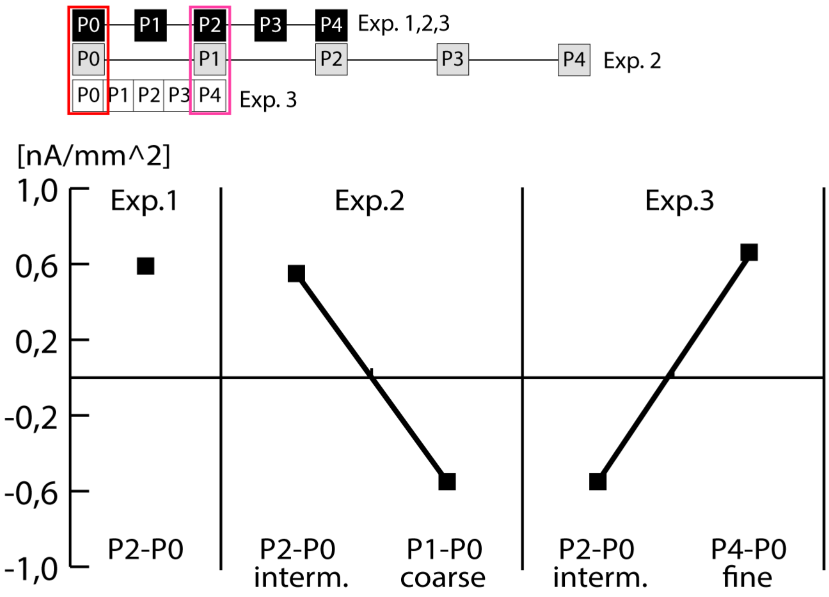

Fig. S4.

Cross-experiment comparison of source activity in higher-level cortex elicited by the same color-combinations. To keep the responses comparable between experiments, we plot the difference of source strength between the one nontarget-color probe (purple box) and the target-color probe (red box) appearing in all experiments. In experiment 1, the intermediate condition of experiment 2, and the fine condition of experiment 3, the response to this probe-color is comparable and larger than the response to P0. This relation, however, changes in the coarse condition of experiment 2 and the intermediate condition of experiment 3. Here the nontarget-color elicits a smaller response than the target-color, illustrating that the relative source activity, elicited by identical color-pairs changes with the overall resolution settings of an experiment.
